# The role of NMR in leveraging dynamics and entropy in drug design

**DOI:** 10.1007/s10858-020-00335-9

**Published:** 2020-07-27

**Authors:** Abhinav Dubey, Koh Takeuchi, Mikhail Reibarkh, Haribabu Arthanari

**Affiliations:** 1. Department of Cancer Biology, Dana-Farber Cancer Institute, Boston, MA 02215.; 2. Cellular Molecular Biotechnology Research Institute & Molecular Profiling Research Center for Drug Discovery (molprof), National Institute of Advanced Industrial Science and Technology (AIST), Tokyo 135-0064, Japan; 3. Analytical Research and Development, Merck & Co., Inc., Rahway, NJ 07065, USA; 4. Department of Biological Chemistry and Molecular Pharmacology, Harvard Medical School, Boston, MA 02115

## Abstract

Nuclear Magnetic Resonance (NMR) spectroscopy has contributed to structure-based drug development (SBDD) in a unique way compared to the other biophysical methods. The potency of a ligand binding to a protein is dictated by the binding free energy, which is an intricate interplay between entropy and enthalpy. In addition to providing the atomic resolution structural information, NMR can help to identify protein-ligand interactions that potentially contribute to the enthalpic component of the free energy. NMR can also illuminate dynamic aspects of the interaction, which correspond to the entropic term of the free energy. The ability of NMR to access both terms in the free energy equation stems from the suite of experiments developed to shed light on various aspects that contribute to both entropy and enthalpy, deepening our understanding of the biological function of macromolecules and assisting to target them in physiological conditions. Here we provide a brief account of the contribution of NMR to SBDD, highlighting hallmark examples and discussing the challenges that demand further method development. In the era of integrated biology, the unique ability of NMR to directly ascertain structural and dynamical aspects of macromolecule and monitor changes in these properties upon engaging a ligand can be combined with computational and other structural and biophysical methods to provide a more complete picture of the energetics of drug engagement with the target. Such efforts can be used to engineer better drugs.

## Introduction

Much like a sculptor transforms a stone into a masterpiece using her artistic tools, a medicinal chemist transforms a molecule in the laboratory setting to a drug in the clinical setting guided by the laws of thermodynamics. Thermodynamic parameters are an important measure used to improve the specificity and affinity of a molecule to given target. Medicinal chemists use a host of biophysical techniques to derive these parameters at different stages of the drug discovery process. Solution state NMR spectroscopy is used in various stages including fragment-based drug discovery (FBDD), evolution of fragments for high affinity binders, lead optimization and structure-based drug developments (SBDD)^1–3^. NMR is used to determine structure of protein-ligand complex, establish binding status, determine the binding mode (ligand and protein atoms involved in direct or indirect binding), ascertain binding specificity, and quantifying the binding affinity^4–6^. A large repertoire of existing and continuously advancing NMR experiments provide quantitative details on the molecular structure, interaction and dynamics at atomic resolution in near physiological conditions^7–10^. NMR can uniquely measure dynamics of the protein upon engaging with a ligand, both from the perspective of the protein and ligand. Furthermore, NMR can provide information about dissociation of bound water molecules, which plays a critical role in the energetics of protein-ligand interactions^11–13^. While most of the efforts in drug design focus on improving the enthalpic contribution, NMR can be used as a probe of conformational entropy, which could be utilized to aid drug discovery. In this perspective, we explore the role of NMR in leveraging entropy in the process of drug discovery.

### Looking under the hood-The energetics of complex formation:

The formation of protein-ligand complex can have several mechanistic steps but can often be abstracted in a simple chemical equation,

P+L↔PL

where P is the free protein, L is the free ligand and PL is the protein-ligand complex. The parameter used to describe the binding strength at equilibrium is dissociation constant (*K*_d_, inverse of association constant *K*_a_),

$$K_{\text{d}}=\left[\text{P}\right]\left[\text{L}\right]/\left[\text{PL}\right]$$

where [P], [L], and [PL] are the molar concentration of free protein, free ligand, and protein-ligand complex, respectively. Lower the value of *K*_d_, higher the affinity of ligand for the protein. For example, a *K*_d_ of 10 nM implies that, at 10 nM concentration of free ligand, half the protein is in bound form with the ligand in ligand excess condition. Typical, potential drug molecules with optimized structures are known to bind to the target protein with *K*_d_ of 10 nM or less. However the binding constants of the initial hits derived from primary screens are typically in the micromolar (high-throughput screening) to millimolar (fragment-based screening) range. Though different types of molecular interactions and motions dictate the *K*_d_ values, it is not evident through the above equation (2). Another thermodynamic parameter that quantitates the strength of binding, the Gibbs free energy, can bridge this gap.

$$\Delta G^{0}=RT\;\text{ln}\left(\frac{K_{d}}{c_{o}}\right)$$

where ΔG^0^ is change in Gibbs free energy upon protein-ligand complex formation at chemical equilibrium (J·mol^−1^), R is gas constant (J·K^−1^·mol^−1^), T is temperature (K) at which ΔG^0^ is measured, *K*_d_ is dissociation constant (mol/L) and c_0_ is set to 1 mol/L to balance the dimensions^14^. The standard pressure (1 bar) is assumed here. A favorable direction of chemical equilibrium is the one which gives negative change in free energy. A lower *K*_d_ signifies negative free energy change in the forward direction (protein-ligand complex formation) in Eq (1). A *K*_d_ of 10 nM would correspond to a change in free energy of −51 kJ·mol^−1^at 298 K. Though this free energy is the sole indicator of how tight the ligand engages the target, it is a cumulative sum of various contributions.

This change in free energy can be further partitioned to contributions from change in enthalpy (ΔH) and change in entropy (ΔS) as denoted in equation (4),

ΔG=ΔH−TΔS

where Δ*H* is change in enthalpy (J·mol^−1^) and Δ*S* is change in entropy (J·K^−1^·mol^−1^). Enthalpy is a convenient term to describe the cumulative effect of internal energy and potential energy, such as energetic gains from hydrogen bond formation, salt bridges, van der Waals interactions and energetic losses from costs of electrostatic repulsion (Pauli repulsion). In statistical thermodynamics change in enthalpy is defined as change in quantity U + pV where U is internal energy, p is pressure and V is molar volume of system. However, in biological context changes in p and V are negligible and change in enthalpy captures change in the internal energy. Entropy is a measure of disorder in the system, which is connected to the change in number of independent microstates as denoted in equation (5)^15^.

**Figure 1.**
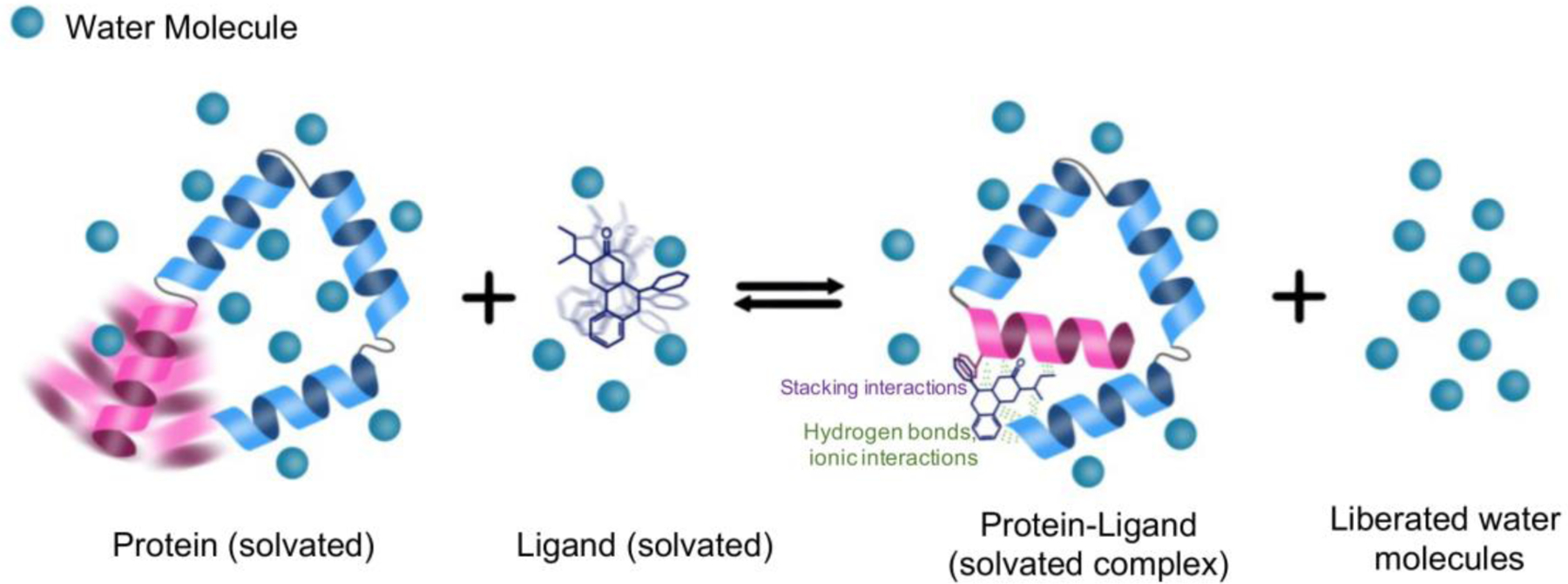
Schematic representation of protein-ligand complex formation. The water molecules (blue spheres) around protein (shown in ribbon) and ligand (stick model) undergo conformational rearrangements upon binding, contributing to entropy and enthalpy changes. The formation of protein-ligand complex also leads to entropic changes due to changes in rotational and translational motion of protein and ligand. The conformation and dynamics of protein can change, as depicted by helix movement (denoted in pink). The protein-ligand complex is stabilized by a number of non-covalent interactions, which contribute to the enthalpy component of the binding free energy. Often, water molecules caged inside protein become free upon binding, making ligand binding entropically favorable.

S=Rln(N)

where *N* is the number of distinct microscopic states available to the system. The equations (3) and (4) taken together provide a blueprint for designing a high-affinity binder. However, in practice these two equations are the holy grail of drug discovery, partly due to the inseparable tango between the two contributions often referred to as enthalpy-entropy compensation. A number of quasi-independent contributions dictate the energetics of protein-ligand complex formation, which can either contribute towards enthalpy and/or entropy changes (Fig. 1). A brief discussion of these contributions is presented here.

### Key players that dictate the energetics of protein-ligand interaction:

The hydration shell around protein and ligand changes upon the complex formation and a new hydration shell envelops the protein-ligand complex. Commonly, the number of water molecules needed to solvate the free protein and ligand individually is larger than the number of water molecules needed to solvate the complex. Thus, the complex formation liberates water molecules, which contributes favorably to entropic component of the free energy equation. In case of protein-ligand interactions, the major contribution to entropy stems from desolvation of the ligand upon binding, especially in case of ligands harboring hydrophobic groups^16,17^; however, this would largely depend on the biophysical nature of the interaction surfaces. In some cases, dissociation of water molecules that were involved in a hydrogen bond to free protein or ligand, will result in an enthalpically unfavorable contribution to the free energy. Concurrently, the protein and ligand can form additional hydrogen bonds, ionic interactions, and van der Waal interactions in complex, which will contribute favorably towards the change in enthalpy. Freitas et. al. performed statistical analysis on ~11,000 structures of protein-ligand complexes and ranked non-covalent interactions based on the frequency of occurrence as follows: i) hydrophobic interactions, ii) hydrogen bonding, iii) π stacking, iv) weak hydrogen bonding, v) salt bridges, vi) amide stacking and vii) cation-π interaction^18^. The hydrophobic interactions were 50% more frequent than hydrogen bonding, the second most prevalent interaction^18^. However, to get a complete picture of the role each interaction plays in free energy, one needs to take into account the strength of each individual interactions. The change in protein and/or ligand structure and dynamics, upon the complex formation, will also contribute to change in enthalpy and entropy, respectively. Several cases are reported where ligand-induced change in protein dynamics at a distal site dictates the mechanism of action. In summary, change in free energy, which defines the binding affinity, is the net result of an intricate ballet between multiple factors, some contributing favorably to the binding event, and others opposing the binding.

### Challenges in obtaining individual estimates of the factors that contribute to the binding free energy:

Experimentally, ΔG is relatively easy to estimate from binding isotherms and equation (3). However, parsing the contribution from enthalpy and entropy terms is usually not straightforward. The total entropic contribution is usually measured indirectly through free energy and enthalpy measurements. Traditionally the van’t Hoff equation has been utilized to derive enthalpy and entropy from free energy. However, in the case of protein-ligand interactions, the linearity of the van’t Hoff equation needs to be carefully assessed to account for the instability of proteins across range of temperatures. Alternatively, Isothermal Titration Calorimetry (ITC) can directly measure both free energy change (ΔG) and enthalpy change (ΔH) upon the complex formation, from the concentration dependent generation and absorption of heat. With equation (4), the entropic contribution can be calculated at a given temperature. However, calculated entropy does not reflect a single phenomenon. As discussed above, ΔS contains contributions from desolvation, protein/ligand conformational changes, ligand translational entropy, and rotational entropy etc. There are empirical methods to estimate desolvation entropy, however, direct measurements remain a challenge^19^. A substantial part of the effort in drug discovery is dedicated to reengineering the drug, using the playbook of thermodynamics, to obtain a more favorable binding free energy. The challenges of parsing out the individual contributions in the binding free energy creates a blind spot in the drug discovery and optimization process. We acknowledge that a large body of work has been conducted in the 1970s–90s and after, to attempt to partition the free energy changes associated with binding. Despite these efforts, this problem is not easily solvable even now^20–24^.

**Figure 2.**
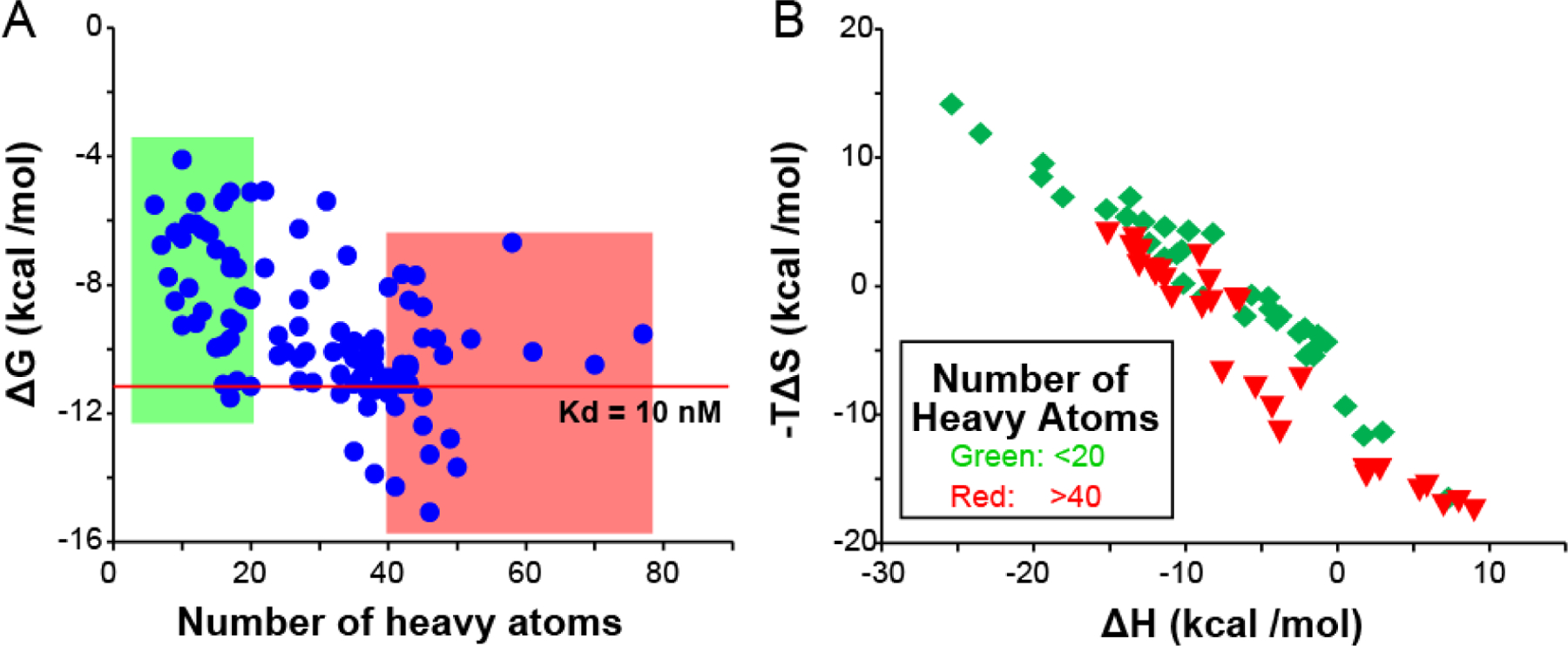
Enthalpy-Entropy compensation in ligand binding. (A) Relation between ΔG and size of molecule. Larger compounds tend to have high affinity compared to smaller compounds. The data were extracted from Reynolds et.al.^29^ (B) Relation between enthalpy (ΔH) vs entropy (−TΔS). Data for small and large (number of heavy atoms < 20 and >40, respectively) compounds that corresponding to the colored shadow in (A) are shown. Enthalpy-Entropy compensation is clearly seen by the anti-diagonal distribution of binding isotherms. The tendency is true for both small and large compounds.

There are number of cases reported in literature where thermodynamically favorable enthalpic change compensates for the loss in entropy to drive the protein-ligand complex formation or *vice versa*. The energy associated with hydrogen bonds is highly variable and is estimated to be in the range of 6–30 kJ/mol^25–27^. However, the formation of the hydrogen bonds occurs at an entropic cost, due to the restriction of rotational, translational and conformational motion of a ligand, estimated to be around 15–25 kJ/mol^25,28^. In the context of protein-ligand interactions, formation of a hydrogen bond may not be an isolated event, as it requires breaking hydrogen bonds with water. Though the desolvation may be entropically favored, removing the hydrogen bonded water on the surface of protein and/or ligand can be enthalpically unfavorable. Examples of such a compensatory relationship between enthalpy and entropy is seen in the binding isotherms in the database^29,30^ (Fig. 2). Increasing the number of favorable interactions to gain a lower dissociation constant (*K*_*d*_) is, indeed, very challenging. In addition, the current SBDD often relies on the static structures, where enthalpic contributions are still difficult to estimate with high accuracy and no information about entropic contributions is included. The enthalpic contribution can be obtained from quantum-mechanical calculation such as fragment molecular orbital method^31–33^. The problem with the entropic term can be circumvented by molecular dynamics simulations, but these estimations harbor errors on the order of ~5 kJ/mol^34–36^, which corresponds to ~10 times difference in *K*_d_ values at ambient temperature. Further, some of the dynamics associated with protein-ligand interactions are in high microsecond to millisecond time regime, not accessible to conventional molecular dynamics simulations. Therefore, a strategy, to not only capture a high-resolution snapshot of the static part of the molecular interaction, but also extract quantitative information on dynamic behavior of protein-ligand complex is needed. Especially if there are lowly-populated, yet therapeutically advantageous minor conformers. NMR spectroscopy has the unique strength to access the both desirables, albeit with some limitations. NMR spectroscopy, in combination with molecular dynamics simulations can measure the proxy for entropy in protein and ligand interactions. Here we discuss the role of NMR spectroscopy in determining the individual contributions to the binding free energy, facilitating drug design. For the sake of clarity, we will separate the discussions into two broad sections, energetics from the perspective of the i) ligand and ii) protein, though in practice they are intertwined.

### Spot-light on change in dynamics upon formation of protein-ligand complex:

Designing a ligand that shows high affinity and specificity to a protein of interest is not straight forward. However, NMR analysis of protein-ligand complex provides a unique opportunity to obtain both the structural information that mainly reflect enthalpic change (ΔH) and the dynamics information that reflects entropic change (ΔS). Before we look at dynamics from a ligand perspective, let us discuss the “breathability” and accommodative capacity of the protein that enables ligand dynamics.

The role of NMR in SBDD is evident from many break-through medications developed through use of NMR-derived structural information. A hallmark example is the development of venetoclax, ABT-199, a therapeutic for chronic lymphocytic leukemia (CLL). This drug inhibits the activity of B-cell lymphoma-2 (Bcl-2), an anti-apoptotic protein by orthosterically inhibiting the protein-protein interactions to the partner proteins. The early development of the Bcl-2 inhibitor was done by fragment-based screening using NMR^37^. The linking of two sub mM affinity fragments based on the NMR-derived structure information successfully improved the affinity of the compound. In addition, a further optimization of ligand based on the structural information led to the development of ABT-199 (FDA approved in 2016), which shows improved specificity to Bcl-2 over the structurally similar protein, Bcl-xL^38^.

It should be noted that some of these potent inhibitors of Bcl-2 and its family of proteins, including ABT-199, cause the opening of a pocket that is not evident in the unbound structure. These pockets, referred to as “cryptic sites”, offer an additional surface interaction site, induced by binding to the ligand, and are found mostly by serendipity. Without a time consuming experimental or computational search for such sites in each target protein, these cryptic sites would be challenging to find^39–41^. In line of this notion, Lee, et al., reported an interesting strategy to identify the cryptic site, which corresponds a lowly populated minor conformation, by using NMR^42^. While investigating inhibitors of LpxC, a novel antibiotic target, researchers found that the inhibitor accesses an alternative conformation of the protein (lowly populated/minor-state) in solution in addition to the major conformation observed in crystal structures. The minor-state conformation utilizes a cryptic interaction site on the protein. They then designed a novel inhibitor that targets the cryptic site to better incorporate the new interaction surface. This strategy led to the development of a potent antibiotic with improved activity, by 2- to 25-fold, relative to the original compound. The results clearly indicate the potential of dynamics information about minor-states accessible by NMR, to improve the efficacy of ligand. Identifying the lowly populated druggable conformations in the target proteins, where binding pocket can be extended, offers great promise.

### Utilizing information about ligand dynamics and desolvation for drug development:

In drug discovery efforts, the potency of compounds needs to be increased while optimizing and maintaining other physicochemical and pharmacological properties to retain drug-like characteristics. It has been suggested that ligands with more enthalpic contribution in binding should be selected as the starting points for lead optimization^17,43^. However, enthalpies of binding only weakly correlates with binding free energies^44^, primarily due to enthalpy-entropy compensation^29^. Interestingly, the HIV-1 (Human Immunodeficiency Virus) protease and aldose reductase appear to have better-than-average correlation between enthalpies and free energies of binding^29^, which may explain why rational drug design has had unusually high success in these targets^45–47^. This suggests that understanding entropic contributions to the binding is very important for successful rational drug design.

Alteration of ligand conformational space upon binding to the protein is one of the sources of enthalpy-entropy compensation. The conformational ensemble sampled by the free ligand in solution is typically different from a bound bioactive conformer. Since the binding to a protein imparts a thermodynamically favorable enthalpic change, this could compensate for any unfavorable conformational “strain” on the ligand, induced upon binding. The concept of “strain” refers to a high internal energy of the bound ligand compared to the free ligand. Enthalpic penalties from the conformational strain are extensively studied and have an average of 17 kJ/mol, with 10% of the cases exceeding 38 kJ/mol^48^. Since NMR is powerful in determining the solution conformation of wide range of ligands, it can be utilized to optimize the free-state ligand conformation to adopt a conformation similar to the bound-state. Prearrangement, which refers to designing the ligand to mimic the bound conformation, allows avoiding both conformational strain and conformational entropy loss to improve the binding affinity^49,50^. Entropic penalties, however, are more challenging to measure and, thus, are less studied.

The thermodynamics of ligand binding can be experimentally measured through techniques, such as ITC. Some of this data has been archived in the public database, BindingDB^51^. Enthalpy-entropy compensations are clearly observed as a thermodynamic feature of protein-ligand interactions^29^ (Fig. 2). In general, protein-ligand interactions that show more favorable enthalpic change upon the binding display more entropic loss (−TΔS) and vice versa. This can happen for ligands with similar sizes and interacting with similar surface areas on the protein^52^, highlighting the important contribution of dynamics in the interaction, despite the conventional wisdom that conformational entropy is a relatively small contributor to binding free energy relative to the displacement of water molecules. However, Chodera et. al have critically examined the enthalpy-entropy compensation in their review. They argue that the severe enthalpy-entropy compensation reported in literature could be due to experimental limitations in the ITC measurements. Their primary objection in studying enthalpy-entropy compensation is the cost benefit analysis of such studies^44^. On the other hand, Fox et. al argue one should study this compensation effects to design ligands which can break the compensation^11^.

**Figure 3:**
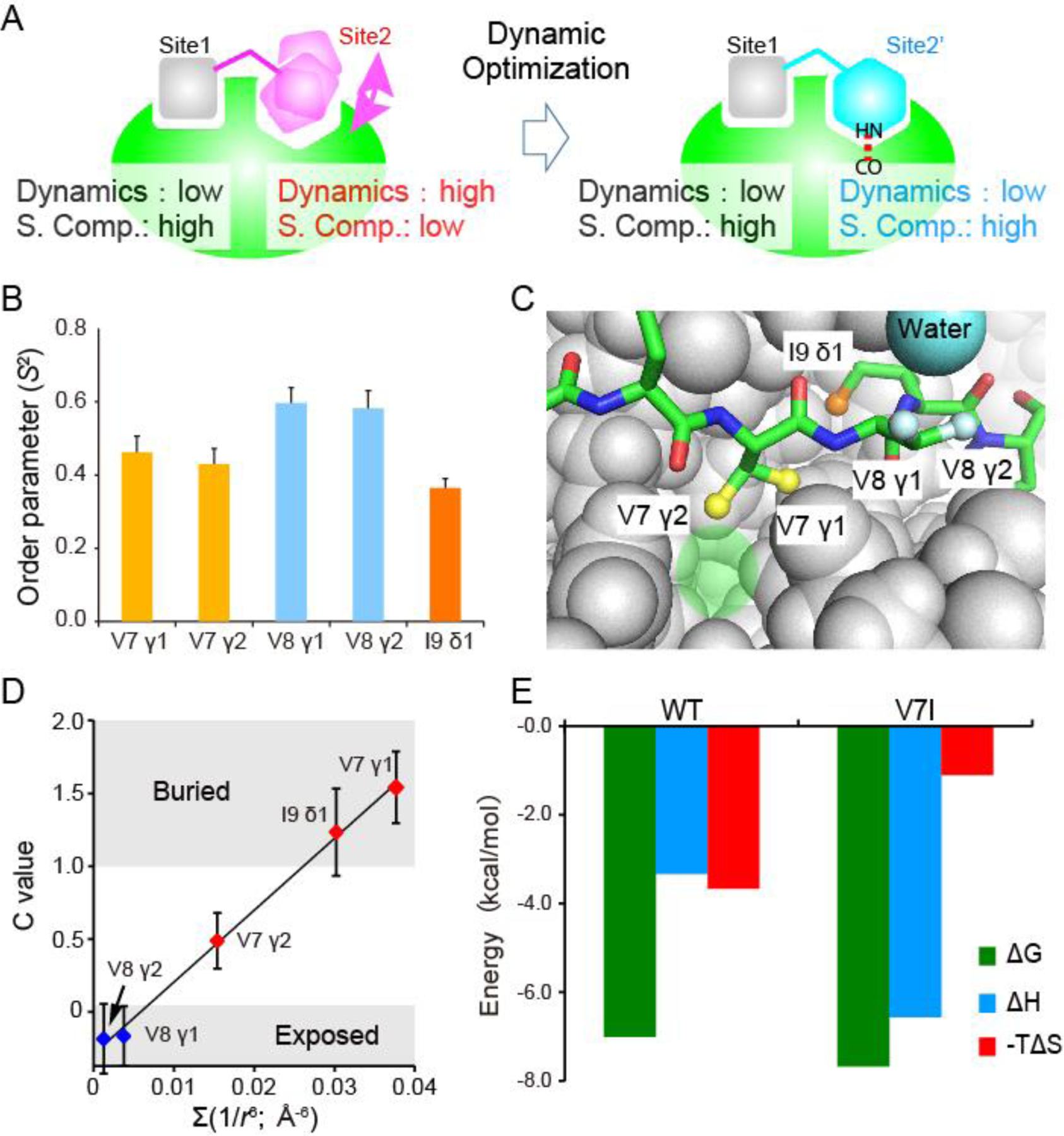
Leveraging dynamics in SBDD by using NMR (A) NMR can ascertain the distribution of dynamics on the ligand to identify the part of ligand that need to optimize. (B) Conformational flexibility of a bound ligand (MEF2A peptide) revealed by NMR. Methyl order parameter (S^2^) values for the MEF2A peptide as determined by forbidden-coherence transfer (FCT) experiments (for details see ref^59^). (C) The structure of the MEF2A docking peptide (stick) in complex with p38α (PDB ID: 1LEW). The methyl moiety in MEF2A peptide are shown as balls with colors corresponding to the bars in (B). The interface methyl moiety retains ns-ps fast dynamics in the bound state. The available space near Val-7 γ2 is shown in green shadow. (D) Correlation between the complementary values (C-value) in the Ex-FCT experiment and the Σ1/r^6^ between the methyl and protein protons, calculated from the structure of the MEF2A peptide-p38α complex (PDB ID: 1LEW). C-value corresponds to number of protons in 2.5Å distance. (E) Binding free energy of the WT MEF2A peptide and the V7I mutant. Figure was replotted from Mizukoshi, Y. et al.^59^

Although ITC and other biochemical/biophysical methods can measure macroscopic thermodynamic parameters, information with greater spatial resolution is required to drive drug design with optimized thermodynamic properties. As NMR is suitable for separately obtaining the dynamics information within different parts of the molecule, the technique can be utilized to optimize a ligand (Fig. 3A). The conformational flexibility and conformational entropy of bound ligands can be determined by Forbidden Coherence Transfer (FCT) analysis^53–55^. For example, using the interaction between the receptor, p38α (a mitogen-activated protein kinase) and a ligand, a myocyte enhancer factor 2A (MEF2A) docking peptide, as a model system, local ps-ns dynamics of a ligand bound to the receptor were directly measured by the FCT analysis. The analysis indicates that methyl groups in the binding pocket of p38α retain substantial dynamics with the order parameter (S^2^) ~0.4, even though the MEF2A peptide is not solvent exposed (Fig. 3B). The FCT analysis can also evaluate the remote proton density around the same methyl moiety by varying the deuteration rate of protein^55^. The analysis shows that there is excess space around the Val-7 γ2 methyl group of the MEF2A peptide when bound to p38 (Fig. 3C and D), for example. Substituting Val-7 with Ile therefore improves the affinity of the MEF2A peptide to WT p38α by 3.1-fold. The thermodynamic parameter of the mutant peptide binding was substantially improved with more enthalpic contributions to the binding free energy (Fig. 3E). Although the enthalpy-entropy compensation is still present, the strategy potentially enables a ligand to have greater enthalpic gains while not losing the conformational dynamics at the local site (*i.e*. no conformational entropy loss). It should also be noted that the FCT method is applicable to ligands that are not ^13^C labeled^56^ as well as ligands containing trifluoromethyl moieties^57^. This example illustrates that FCT experiments that can inform on ps-ns dynamics, in the bound state considering only dipole-induced relaxation, neglecting contributions from CSA (chemical shift anisotropy). This FCT directly provides information on the order parameter S^2^, of bound ligand, which can be used to improve binding affinity.

The uneven distribution of dynamics across interaction surfaces is not only important in dynamics-based ligand optimization but is also critical to understanding the function of proteins, especially those that work as the hub in interactomes. An example of this is the competition between the binding of two unstructured peptides from proteins HIF-1α and CITED2, to bind to a common target protein domain TAZ1, a part of the co-activator CBP/p300. HIF-1α is easily dissociated by the same concentration of CITED2, but not vice versa, even though the binding affinities of these two peptides to TAZ1 are identical. This is explained by the flexibility in neighboring region of the binding motif in the HIF-1α-TAZ1 complex leading to transient and partial dissociation of the dynamic part of HIF-1α, allowing CITED2 to wedge in, and increasing the rate of HIF-1α dissociation^58^. This local competition mechanism and dynamics information can be potentially used to improve the efficacy of a ligand to target a weak dynamic spot in protein-protein or protein-substrate interaction interface. Such a spot can be identified by the ligand dynamics information in the complex. However, this concept is just a speculation at this stage and need to be proven with an experimental demonstration.

### Harnessing dynamics in the microsecond to millisecond time regime for drug discovery:

Measuring dynamics in the μs and ms range using ^1^H R_1ρ_ and R_2_ relaxation dispersion (RD) experiments, respectively, provides important insight into the kinetics of ligand binding. RD experiments provide *k*_ex_ values and with the knowledge of the *K*_*d*_ values one can deduce the kinetic parameters, *k*_on_ and *k*_off_^45^. This provides comprehensive understanding of the protein-ligand interaction that is crucial for drug design and development^60,61^, as illustrated with several examples below.

### The role of thermodynamics versus kinetics in drug discovery:

Given the breadth of techniques at hand, it is possible to characterize protein-ligand interactions using thermodynamic *or* kinetic parameters. While the thermodynamic and kinetic parameters that report on binding are related to each other, they measure different characteristics of the binding event, which is critical to drug discovery. Thorough investigation of both thermodynamic and kinetic parameters is necessary to adequately purpose a drug. For example, the dissociation constant *K*_*d*_ is expressed thermodynamic quantity in equations 4 and 5. *K*_*d*_ can also be derived from kinetic parameters, namely the *k*_*on*_ and *k*_*off*_, the on and off rates:

$$K_{d}=k_{\text{off}}/k_{\text{on}}$$

Thus, two ligands could have the same *K*_*d*_, despite having on/off kinetic parameters that are different by orders of magnitude (5 = 500/100 = 5/1). Both thermodynamic and kinetic parameters constitute unique implications for a given drug’s application, as described in the following examples.

Slow off-rate is often highly desirable, as it increases the residence time, defined as 1/*k*_off_. The importance of the resident time in ligand optimization is now widely recognized in the field of drug discovery^62,63^. As an example, let’s consider ipratropium and tiotropium: two muscarinic M3 receptor antagonists, whose *K*_*d*_ values differ by a factor of 2. Despite comparable *K*_*d*_ values, they exhibit a 200-fold difference in the *k*_off_, which drastically impacts their pharmacological efficacy^64,65^. Tiotropium, owing to the slow off-rate, exhibits long lasting effects with little variation in bronchiodilatation between peak and trough concentrations. Thus, its desirable kinetic parameters rendered it the first once-a-day bronchodilator^48^.

Kinetics can also help define the selectivity of binding to the target. In general, a faster off-rate from unintended targets and a slow off-rate for the intended target is desired. This concept of harnessing a slower off-rate for selectivity was used in the case of neuropeptide Y (NPY). Though NYP had comparable K_d_ for receptors Y2, Y1 and Y5, the slow off-rate upon binding to Y2 imparted selectivity to Y_2_ receptors relative to type Y_1_ and type Y_5_ receptors^66^. This effect is called kinetic selectivity and it is common in drug discovery and lead optimization^67^.

A fast on-rate of the drug is pharmacologically desired in many cases such as antithrombosis drugs. Despite a 50,000-fold difference between the *K*_i_ values (inhibition constant, which is very similar to the dissociation constant *K*_d_), comparable plasma concentrations of drugs hirudin and melagatran were needed to obtain a 50% antithrombotic effect in vivo^68^. These differing profiles have been explained by the concentration of thrombin in the thrombus and the rate of attaining an effective inhibitory concentration of antagonist drug^68^. This study also demonstrates that compounds rapidly reaching critical concentration for efficacy have a better therapeutic index as well^68^.

On-rates are also indicative of the mode of binding, which is a very important information during the early stages of lead optimization. The diffusion limit of *k*_on_ values for small molecules is well understood and is on the order of 10^9^ M^−1^ s^−1 69^. If the *k*_on_ of two small molecule ligands are similar, it indicates minimal conformational rearrangements upon binding and that the binding is diffusion controlled. Contrarily, if the *k*_on_ of a small molecule ligand is three to four orders of magnitude smaller compared to another ligand, it may indicate at the presence cryptic site or other modes of significant and correspondingly slow protein conformational rearrangement upon binding with the ligand with the slower on-rate. For instance, binding of inhibitors to InhA, the enoyl-ACP reductase of *Mycobacterium tuberculosis*, involves conversion of a disordered loop into an ordered a helix at the active site^70^. This large conformational change appears to impact the rate of inhibitor binding and unbinding significantly, with slow-onset and slow-offset inhibition being associated with the ordered helix^70,71^. An inhibitor PT70 was specifically designed to interact with the ordered InhA helix and the NAD+ cofactor.by the addition of a single methyl group^71^. This seemingly small change was found to increase the residence time of PT70 by 4 orders of magnitude compared with triclosan. The crystal structure of the ternary complex between InhA, NAD+, and PT70 demonstrated that slow onset inhibition is indeed coupled to the structural rearrangement of an active site loop of InhA^71^.

As demonstrated by the aforementioned examples, both thermodynamics and kinetics of the ligand-protein interaction are important for successful lead optimization and drug development. Thus, kinetics must be considered in concert with thermodynamic properties. Of note is a current lack of systematic approaches for the design of ligands with optimal kinetic properties^72^. As will be shown below, NMR offers multiple ways to provide kinetic and dynamic characterization. We hope that this review will generate interest in these NMR applications in the drug discovery and development.

### Slow dynamics related with conformational exchange:

A ligand can have slower intrinsic conformational flexibility and change its flexibility upon receptor interaction. Such slow ligand flexibility can be identified by a ^13^C RD measurements at natural ^13^C abundance. Since the RD experiment is sensitive enough to detect ligand populations as low as 1%, bound-state information becomes available for NMR signals of ligands that are exchanging between the major free and the minor receptor-bound states to improve the sensitivity^73^. The RD observed is result of various sources of ^13^C chemical shift modulation, including binding and conformational changes in both free and bound states. This strategy was applied to three structurally similar ligands of human Pin1, a peptidyl-prolyl isomerase, which varied in flexiblility^73^. The ligands include a phosphopeptide substrate, FFpSPR, that can isomerize between the cis-trans conformations at the pSer-Pro (pSP) motif, and two inhibitor analogues that can replace the core pSP motif by alkene isosteres, to conformationally lock the position to cis or trans. The authors successfully identified the site-specific ^13^C RD to the pSP motif in the FFpSPR peptide, while both of the pSP locked inhibitors did not exhibit conformational changes in the RD experiments. Thus, the strategy would identify the locations and time scales of motion on the peptide in the bound form and how this can be perturbed by ligand structural modifications. In addition, bound-ligand conformation can be constrained via ^13^C chemical shift information derived from the fitting of the ^13^C RD data.

Binding dynamics in the millisecond range often lead to an intermediate exchange regime with a consequent undesirable line-broadening. In the drug discovery process, it is characteristic for initial hits or first-generation compounds to have dissociation constants in the mid-micromolar range, resulting in line broadening of the NMR spectra. However, exchange in the intermediate regime is not the only contributor to line broadening, as increased ligand dynamics in the bound state (due to an exchange between multiple binding modes) can cause a similar effect. It is important for subsequent structure-based compound optimization to incorporate an understanding of the genesis of line broadening. An NMR method for distinguishing between intermediate exchange versus conformational heterogeneity, been developed and demonstrated on ^13^C ILV-labeled Bcl-xL^74^, by observing methyl resonances of the protein. Two homologous small molecule ligands of Bcl-xL with mid-micromolar dissociation constants causing severe line broadening of interface residues upon binding were chosen. For the compound that exhibited a single-mode binding, the broadening was solely attributed to the dissociation kinetics in the intermediate exchange regime. Here, the line broadening can be overcome with high molar excess of the ligand. For the other compound, line broadening was due to both dissociation kinetics and an exchange between multiple bound conformations. In this case, the broadening could not be overcome with excess ligand.

As we discussed here, utilizing information about ligand dynamics is quite important to understand the thermodynamic and kinetic nature of ligand and to obtain a ligand with higher affinity, preferable thermodynamic profile, specificity, and resident times for drug development. A suite of NMR experiments that define the dynamic of ligand with wide range of time scale with the moiety-specific resolution is of importance to experimentally quantify and evaluate the dynamics-activity and dynamics-function relationship of ligands.

#### Dynamics and conformational exchange of cyclic peptides as ligands in the context of drug design.

Macrocyclic peptides represent a special therapeutic modality that has a potential to combine the best properties of small molecule ligands (synthetic accessibility, cell permeability) and antibodies (high specificity, ability to target shallow or flat protein surfaces traditionally considered “undruggable”)^75,76^. Significant development in recent years is evidenced by steadily increasing numbers of primary and secondary publications^75^, as well as a number of cyclic peptides entering clinical trials^77^. Conformational behavior of cyclic peptides is quite complex by itself with many degree of freedoms in their structure, and there are still open challenges in the field with understanding and predicting conformation, activity, especially the permeability of cyclic peptides^78^.

Understanding conformational flexibility and corresponding conformational entropy for drug design is even more important for cyclic peptides than for small molecule ligands, and NMR is widely utilized in this space. Macrocyclization imparts a degree of conformational restriction and structural preorganization, which is thermodynamically favorable because this reduces entropic losses - compared to linear peptides - due to binding^75^. The constrained nature of cyclic peptides creates a challenge when a relatively small structural modification results in significant conformational reorganizations of remote regions of a macrocycle^79^. One the other hand, cyclic peptides can access vast conformational space with some non-canonical conformations^80,81^. The conformational strain tolerated by therapeutically-relevant macrocyclic peptides upon target protein binding is postulated to be two to three times higher than that of small molecule ligands discussed above^82^. Apart from stapled peptides with rigid structures mimicking the protein-protein interaction^83^, it is believed that a typical macrocyclic peptide will access complex conformational space in solution and adopt a significantly different conformation in the bound state^84–90^. In a study that jointly utilized NMR, crystallographic and computational approaches, the importance of conformational dynamics for understanding and optimizing binding to the target protein is exhibited. Conformational complexity of cyclic peptides presents a challenge for computational methods as well, since near-exhaustive conformational search is needed, but difficult to achieve with desired precision. Several recent reports demonstrate the utility of NMR parameters such as chemical shifts^91^, coupling constants and sparse NOEs^92^ to augment computational protocols either as explicit restraints (NOEs and coupling constants) or to provide an orthogonal validation (chemical shifts, residual dipolar couplings).

Conformational dynamics of cyclic peptides have additional implications for another key determinant of drug uptake, passive cell permeability. Unlike small molecule drugs, cell permeability is not easily achieved, yet critical, for therapeutic macrocyclic peptides with intracellular targets. A classic example is the natural product cyclosporine A, a cyclic undecapeptide immunosuppressant that is passively permeable, leading to high oral bioavailability^93^. Passive permeability of the cyclosporine A is attributed to a dynamic conformational interplay between an aqueous conformation, a membrane-preferred conformation and a protein-bound conformation^94,95^. Similar mechanisms have been described for cyclic peptides of natural product^96^ and synthetic^97^ origin. Behavior of cyclosporin A is often used to rationalize membrane permeability differences for a series of related peptides^98^. However, it is still not clear whether this mechanism of permeability is typical or not for cyclic peptides in general. It should be noted that non-peptidic macrocycles are also pharmaceutically important molecules^99^ with complex conformational dynamics. Recently, ^1^H and ^13^C RD has been shown to characterize conformational dynamics (on- and off- rates and transition barrier) of an a-cyclodextrin^100^.

However, rational engineering of passive permeability remains a sizeable challenge in the field and we envision that NMR, with its ability to study conformations and dynamics of cyclic peptides in both aqueous and in lipophilic environments, will contribute to addressing this challenge.

#### Importance of fast protein fluctuation: source of conformational entropy

**Figure 4.**
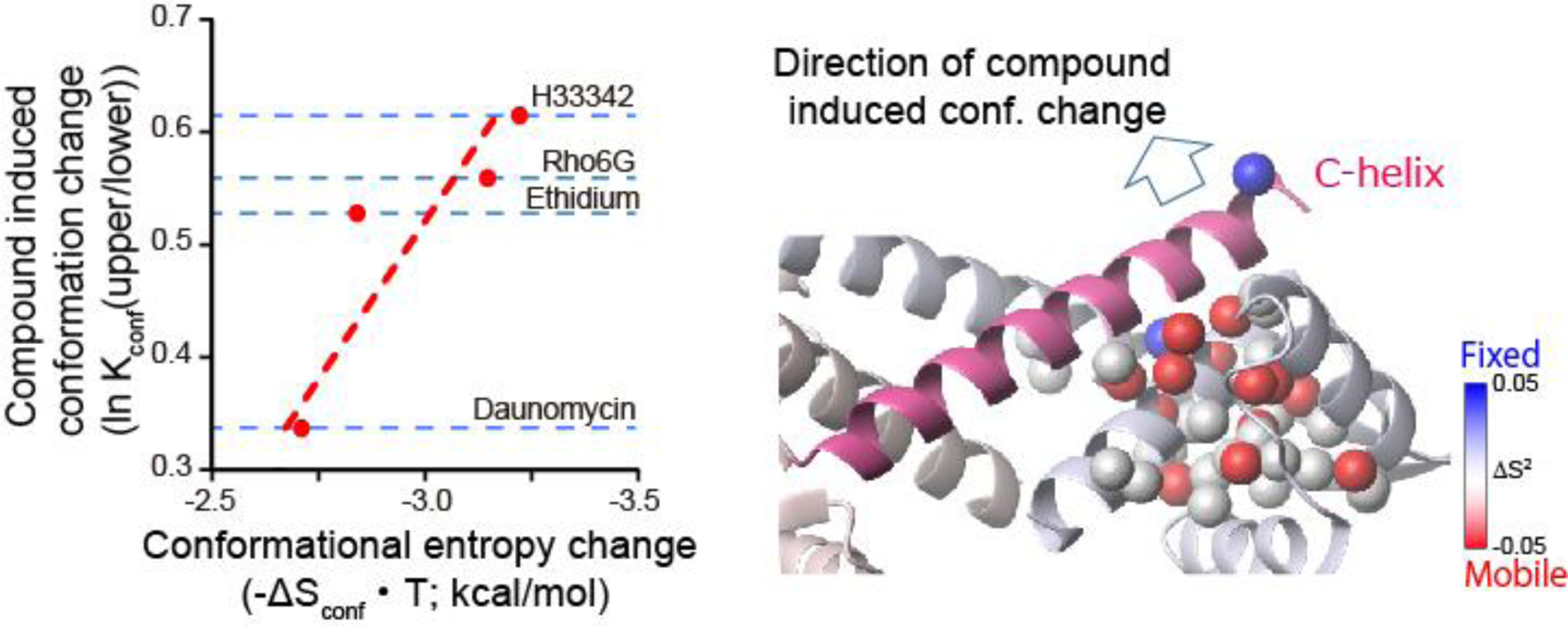
Importance of dynamics in protein-ligand interactions. (A) The conformational equilibrium revealed by NMR explains the conformational entropy gain coupled to binding a ligand in multidrug binding transcription factor, LmrR. The population shift upon ligand binding correlates with the conformational entropy gain calculated from the changes in fast methyl dynamics (for detail see ref^113^) (B) The coupling between compound binding and enhancement of methyl dynamics in hydrophobic core. The upper displacement of the C-helix caused by compound binding loosens the hydrophobic core beneath the helix.

In addition to the conformational entropy of the ligand, the contribution of receptor conformational entropy has been shown to be important. Changes in protein conformational entropy upon binding can be estimated from experimentally determined order parameters^101–107^. Empirically, the changes in the fast dynamics of methyl-bearing amino acids can be used as the “entropy meter” to estimate the changes in conformational entropy of the protein systems upon the binding to ligands^108,109^. In the case of calmodulin-peptide interactions, it has been shown that the conformational entropy of calmodulin, estimated from the changes in fast methyl dynamics, explain the variety in entropic contribution of the interactions. The contribution of the conformational entropy to the interactions are estimated to be of the order of −30 kcal/mol^107,110^. In case of the carbohydrate recognition domain of galectin-3, the gain in conformational entropy is comparable in magnitude to the binding enthalpy of a ligand^111^. It has also been shown that a large conformational entropy gain makes a mutant of the Catabolite Activator Protein (CAP) bind DNA, although the protein resides mostly in an inactive conformation and needs to confer larger enthalpic energy penalties to adopt the DNA bound conformation^112^. The enhancement of dynamics upon the binding to compounds is considered to contribute to the promiscuous interaction of a multidrug-binding transcription factor, LmrR^113^ where the promiscuity is a part of the function (Fig. 4A). We have shown that compounds binding to LmrR resulted in a notable increase in the amplitude of ps-ns dynamics at the hydrophobic core of the protein, which is allosteric to the compound-binding interface but connected to a ligand binding helix, C-helix (Fig. 4B). It should be noted that the compound-induced change in the C-helix conformational equilibrium correlated with the amount of the conformational entropy gain upon the binding to the compound (Fig. 4A). This implies that displacement of the C-helix caused by compound binding loosens the hydrophobic core beneath the helix (Fig. 4B). The conformational entropy associated with the enhancement of dynamics corresponds to 2–3 kcal/mol, which favorably contributes to its function as a promiscuous sensor for toxic compounds. It should be noted that an increase in conformational entropy is not a common occurrence. In general, a decrease or no significant change in conformational entropy of the binding partners is observed. However, the examples discussed here indicate that conformational entropy should not be neglected in the understanding the thermodynamics of interactions.

#### Importance of slower protein conformational dynamics in ligand specificity and kinetics.

Though the development of highly selective inhibitors is desired in most cases, the process of achieving this has been challenging. This is especially true among a family of proteins that can bind to the same endogenous substrate. Protein kinases are one such major drug targets. Due to the similarity of their active sites, the development of an inhibitor specific for a single kinase is considerably difficult. Nevertheless, there are inhibitors that are more specific than others. *Kern* et al suggested that specific binding pair of protein and ligand show higher affinity due to the ability to change conformation after binding^114^. A representative example of this is the kinase inhibitor Gleevec. Although the crystal structure of Gleevec in complex with a potent and specific target, Abl kinase, is identical to how Gleevec engages the Src kinase, the compound shows affinity to Abl that is 3 orders of magnitude stronger than the affinity to Src kinase. Using NMR chemical shift perturbation and stopped flow experiments, the authors showed that Gleevec has a fast binding followed by a slow conformational change. The latter phase was not observed in the binding to Src kinase. The presence of induced-fit mechanism after binding to the specific ligand were also true for Aurora A kinase inhibitors^115^. These examples clearly indicate that the dynamics in the bound state might be a critical factor to define affinity, specificity and resident time of a ligand.

The importance of protein dynamics is also evident in drug resistance mutations. *Saleh* et al showed that the allosteric drug resistance mutations found in the Abl regulatory module (RM) and far from the drug-binding site alter the population of inhibited and activated states in the Abl kinase. These mutations increase the activated-state population and overall kinase activity that compromise the inhibitory activity of imatinib, without interfering directly with imatinib binding^116^, representing a dynamic mechanism of development of drug resistance.

In dihydrofolate reductase (DHFR), the binding of two drugs, methotrexate and trimethoprim to the holoenzyme (NADPH complex) has been shown to decouple the protein dynamics in an allosteric manner^117^. Upon the binding to these drugs, the major conformation of the NADPH complex did not change, however, its dynamics change in the presence of ligand. The binding of these drug results in either quenching or slowing down of ms-μs conformational exchange in the functional loops and substrate-binding site, working as the molecular straitjacket. In addition, drug binding also causes significant changes in ps-ns dynamics, especially in the distal F-G and adenosine binding loops that contain residues implicated in DHFR function. As these residues that experience a change in dynamics are not in contact with drug. Here the mechanism of inhibition by the small-molecule is by modulating the dynamics at an allosteric site, which can be deciphered by NMR.

Hidden allosteric couplings between two functional sites are not unique to DHFR; we also showed functional coupling between allosteric substrate docking sites and ATP binding site in MAPK, p38α^118^. It should be noted that the protein dynamics in ligand bound form is considered to be one of the determining factors in determining the dissociation rate of a ligand, nicely depicted by RD NMR experiment^119^. Therefore, it is evident that the slower protein conformational dynamics, which is essential for function, is also a good target for therapeutic intervention. Although the specific path to achieve this goal needs to be experimentally explored and could depend on the target and the flavor of dynamics available, this slow protein dynamics can be leveraged to in obtain ligands with better affinity, specificity, and/or favorable binding kinetics. These slow millisecond dynamics can be observed by NMR methods as the major conformational state can be identical and cannot be distinguished by other structural methods.

## NMR methods to study dynamics and conformational exchange

The static picture of protein-ligand interactions is obtained through solving the structure of the complex using X-ray crystallography and NMR spectroscopy. Insights on conformational changes can be made by comparing protein structure in both bound and free form. Additionally, NMR can give site-specific exclusive insights for dynamics at multiple time scales and range of binding affinities. Measurements of transverse relaxation rate (R_2_), longitudinal relaxation rate (R_1_) and 2D ^15^N-^1^H heteronuclear nuclear Overhauser effect (het-NOE) on ^15^N/^13^C isotopically labeled protein; coupled with Lipari-Szabo model free analysis can provide dynamics information at pico-nanoseconds timescales^120,121^. *Wand* et. al have proposed Lipari-Szabo squared generalized order parameter of methyl bearing side chains as a dynamical proxy for overall protein conformational entropy and have empirically calibrated the ‘entropy meter’ on 28 protein-ligand complexes with a broad range of binding affinities^106^. In addition to these strategies, the FCT method has been proposed, to estimate the ps-ns dynamics of methyl groups.^53,122^ In the FCT experiment, the intensity of the multi quantum methyl proton signal (*I*_mqc_) relative to the intensity of the corresponding single quantum methyl proton signal (*I*_1qc_) is measured. The evolution of *I*_mqc_/*I*_1qc_ can be described by a formula that consists of methyl order parameter (*S*^2^) and the dipole cross-correlated relaxation rate to external protons, respectively^122^. The FCT experiment can be combined with the methyl TROSY (transverse relaxation optimized spectroscopy) experiment to quantify the dynamics of large molecular weight systems such as a 360 kDa “half-proteasome” complex^54^. The applicability of the FCT method to high molecular-weight (*M*_W_) proteins is attractive in structure-based drug developments, as the proteins of interest tend to be large in *M*_W_ (> 40kDa).

Relaxation dispersion experiments can provide insights into motion on the micro-milliseconds timescale to capture domain rearrangements upon ligand binding. The two methods to measure relaxation dispersion are (a) R_2_ relaxation dispersion using a CPMG (Carr-Purcell-Meiboom-Gill) pulse train and (b) R_1ρ_ relaxation dispersion using spinlock^120,121,123^. Both the methods quantify the linewidth broadening due to chemical or conformational exchange between ground and excited states. Along with the assumption of a kinetic model, one can derive structural, kinetic and thermodynamic information of ground and excited states with this class of experiments. Structural information is obtained through the chemical shifts of the two states, kinetic information is calculated as we get the exchange rate between the two states and by measuring relative population of the two states, we get thermodynamic information.

Dark-state Exchange Saturation Transfer (DEST)^124,125^ and Chemical Exchange Saturation Transfer (CEST)^126,127^ represent another set of NMR experiments to study lowly populated states, also referred to as ‘invisible’ states. This is accomplished by detecting the NMR signals of visible (detectable) state after perturbing the invisible state. Results from CEST and DEST experiments partly complement data obtained from relaxation dispersion experiments. We can use DEST to study exchange dynamics in timescale of 10 ms to 1 s between bound and free form of protein-ligand complex^124^. The linewidths in bound form (dark-state) is broadened beyond detection limit and is saturated with weak radiofrequency (rf), which leads to intensity reduction in visible stat with detectable linewidth. CEST also works on similar principle but it relies on chemical shift difference between the two states rather than difference in linewidths as in case of DEST. With CEST one can readily detect the chemical shift of excited state species with fractional population of excited state is greater than ~ 0.5% and its lifetime is between 3 to 50 ms^126^. Thermodynamic and kinetic information can be extracted from both DEST and CEST experiments using Bloch-McConnell equations to assumed two or three state exchange models. In the context of drug discovery these experiments can be used to detect minor confirmations of the protein including cryptic sites and obtain information about the bound state if the binding results in line broadening beyond detection.

Hydrogen-deuterium (HD) and ZZ exchange experiments are useful to study slow binding events on the timescale of milliseconds to seconds^121^. In contrast to relaxation dispersion experiments, the requirement of chemical shift difference, for these experiments, between the free and bound form is relatively small. The loss in intensity due to exchange of amide protons (^1^H) with ^2^H quantifies the exposure of protein residues to solvent.

Above described NMR experiments covers dynamics spanning wide range of timescales, which can give valuable insights in entropically optimizing the protein-drug affinity.

### NMR methods to study desolvation:

As noted earlier desolvation, especially desolvation of the ligand is major entropic contributor to the binding free energy. Dunitz estimated a range of 0 to 27 J mol^−1^ K^−1^ for the entropic cost of transferring a water molecule from solvent to a protein, leading to ~ 0 to 8 kJ/mol of entropic cost to the free energy^128^. However, there is a large range in the contribution of desolvation to the free energy, which partly depends on how tightly the water molecule was initially bound to the protein. Typically, when a ligand binds to the protein and replaces a bound water molecule, the hydrogen bond between the water and the protein will be replaced by a hydrogen bond between the ligand and the protein, thus compensating for the enthalpic loss. However, the liberated water molecule contributes favorably towards entropic component of the binding free energy. Bound water molecules have various residence times and the tightly bound water molecules are seen in high-resolution X-ray structures. In NMR experiments, bound water and labile protons on the protein surface with lifetimes longer than sub ns can be identified by the NOESY (Nuclear Overhauser Effect Spectroscopy) and ROESY (Rotating frame Overhauser Effect Spectroscopy) experiments^129^. For example, four internal water molecules seen in the crystal structures of bovine pancreatic trypsin inhibitor (BPTI) gave NOEs to protein indicating that the waters are an integral part of the solution conformation. The strategy was further corroborated by using a reverse micelle to avoid the artificial effect from bulk water^130^. It should be noted that by performing WaterLOGSY (Water-Ligand Observed via Gradient Spectroscopy) experiments^131^ at increasing concentrations of per-deuterated proteins are used to identify the existence and location of transiently bound water molecules in protein ligand complexes^132^. In the experiment, the titration slopes can be used as a measure to the primary candidates for substitution with groups that either displace or address protein-bound water molecules^132^.

### Structure determination of the protein-ligand complex using NMR:

The co-structure of the ligand bound to the protein provides vital structural information that drives medicinal chemistry efforts. Though X-ray crystallography plays a significant role in the structure elucidation, not all protein-ligand complexes can be crystallized. In a number of cases, NMR has been used to determine the structure of the complex using distance restraints derived from NOESY and PRE (Paramagnetic Relaxation Enhancement) experiments. It is often a challenge to parse out the intramolecular NOEs from the intermolecular NOEs. Filtered NOEs^133^ experiments that distinguish signals from labeled proteins to unlabeled ligands are often used to obtain unambiguous constraints. In addition, anchoring PRE probes, at specific locations on the protein, acts a GPS to triangulate distances^134^. In the case of intermediate exchange and a corresponding undesired line-broadening, driven NOEs^135^ can be used to obtain distance restraints where peaks are too broad for NOESY. For weak binders in the fast exchange regime, transferred NOESY experiments^136,137^ can be used to determine the bound conformation of the ligand by analyzing the difference between NOE patterns in the absence and in the presence of the protein. Anisotropic NMR, mainly RDC (Residual Dipolar Coupling)^138^, is a valuable structural method as it provides orthogonal restraints, which are global in nature as opposed to inherently local restraints from NOEs and PREs.

Certain proteins, owing to their large size, flexibility and difficulty to crystallize are not amenable for structural studies using solution NMR spectroscopy or X-ray crystallography. Cryoelectron microscopy (cryo-EM) has gained prominence as powerful structure determination technique capable of handling intractable proteins including therapeutically important membrane proteins like GPCRs^139^. Recent technological advances in cryo-EM the structures of protein/protein complexes of size ~100 kDa or more with resolution of below 3 Å^139,140^. Recently a cryo-EM structure of apoferritin was reported at a resolution of 1.25 Å, providing a glimpse of resolution that be routinely obtained in the future^141^. For SBDD and fragment-based screening, high-resolution structure determination and the high-throughput nature of the technique are important requirements. Saur et.al. have highlighted the utility of cryo-EM for SBDD and fragment-based screening using Bgal, a 465 kDa homotetramer protein from E. coli and oncogenic PKM2, a 240 kDa homotetramer as examples^142^. Though cryo-EM currently does not provide detailed information about the dynamics, like NMR, there are efforts to extract such information from the variability in the structural ensembles observed in cryo-EM^143,144^. Structural data from cryo-EM and dynamics data form NMR can be synergistically combined to provide a richer picture of structure and dynamics, combining the advantages of both techniques^145^.

Determining the structure of the protein-ligand complex provides a critical aspect of our thermodynamic understanding of ligand engagement, revealing information on the bound state/conformation of the protein/ligand and any associated change in dynamics.

### Current challenges:

#### Methods for small molecules in the absence of labeling.

Isotopically labeling small molecule ligands is typically difficult and expensive. In addition, small molecule drugs are becoming more structurally complex and more proton deficient. The field of small molecule NMR has seen a significant effort in pulse sequence development^146,147^ to measure critical structural and dynamic parameters on ^13^C and ^15^N natural abundance. The ^2^J and especially ^3^J ^1^H-^1^H, ^1^H-^13^C, ^1^H-^15^N and ^13^C-^13^C coupling constants are critical for characterizing both conformation and dynamics of the respective sites. Pulse sequences such as EXSIDE (Excitation-Sculptured Indirect-Detection Experiment)^148^, IPAP-HSQMBC (in-phase anti-phase Heteronuclear Single Quantum Multiple Bond Correlation)^149,150^ and J-modulated ADEQUATE (Adequate sensitivity Double Quantum Transfer Experiment)^151,152^ are used to measure these coupling constants. Anisotropic NMR methods such as RDC and RCSA (Residual Chemical Shift Anisotropy) have been shown to be useful for structure and stereochemistry elucidation of small molecules^153^. They can also be used to assess conformational dynamics of small molecules^154^. RCSAs are particularly useful for small molecules as they are measured for ^13^C nuclei and interpreted in conjunction with DFT (Density-Functional Theory) calculations, obviating the need for the presence of ^1^H nuclei. These are recent developments and we hope that NMR conformational studies of small molecules will become a standard requirement in the drug discovery process.

Advances in medicinal chemistry allow incorporation of fluorine atoms relatively easily^155^. Fluorine is frequently introduced in the lead optimization stage to enhance affinity, bio-availability, and metabolic stability^156^. Approximately 25% of therapeutic drugs on the market contain at least one fluorine atom^157^. As ^19^F is the 100% naturally abundant 1/2-spin nucleus with the next largest gyromagnetic ratio to the hydrogen and has large chemical shift distribution (~100 ppm), ^19^F-NMR is an attractive approach in drug discovery^158^. Of note, CF_3_ moiety has been extensively used in drug-screening procedures as well as the characterization of drug-target proteins due to its high sensitivity and relatively narrow line width^159–163^.

### Open questions in the field:

Though NMR can provide details about dynamics, conformational changes, desolvation and structural information on how a drug engages the ligand, a methodology to leverage/optimally utilize this information to design better binders is not straightforward. What should be optimized, enthalpy or entropy is a key question, which is further complicated by their interdependence. One of the well-travelled paths is to reduce the conformational space of the ligand by making the ligand resemble the bound structure, thus reducing entropic costs. Even for this logical viewpoint, however, there may be the following downside.

### A tight fit may not be the best fit:

Proteins are inherently dynamic and enthalpy-optimized tight-fitting drugs may not always be the optimal choice. Fernandez et. al makes a compelling case and argues for loosely fitted drugs which allow the target protein to explore the conformational space upon binding^164^. In this case, the polar groups on ligand also explore conformational space on target protein to compensate the thermodynamic cost incurred by losing its hydration shell upon binding. The two anticancer inhibitors sunitinib and sorafenib are the kinase inhibitors for the target proteins C-Kit and p38 mitogen activated protein (Map) kinase, respectively, which have hydrophobic-polar mismatch at the protein ligand interface^164^. The hydrophobic-polar mismatch is discouraged from enthalpic point of view, while the significant conformational entropy gains explains their observed high affinity. It is therefore advised to keep conformational entropy factor in drug design strategy for highly dynamic protein targets. Avoiding tight fitting binders have been shown to be beneficial to cope with mutation-induced drug resistance developed by pathogens. For example, frequent mutations are reported in Reverse Transcriptase of HIV-1 which confers resistance to nucleoside/nucleotide analogs (NRTI) and non-nucleoside (NNRTI) analogs^165,166^. Usually, mutant proteins evolved with drug resistance are the ones, which create steric hindrance for drugs where the drug used to perfectly bind. This drug resistance mechanism can be thwarted by allowing conformational flexibility on the ligand. With the capability of NMR to access conformational dynamics, we have the ability to pursue an alternative path breaking away from the dogma of designing tight and rigid binders. Flexible ligands that may have greater versatility in some cases.

### Choosing the sweet spot between hydrophobicity and solubility:

A common approach in optimizing ligands for potency and selectivity is to focus on design principles that increase the enthalpic contribution, especially if high-resolution structure is available. Freire argues that it is much more difficult to optimize a ligand for enthalpy gains, primarily because the gains in enthalpy can be compensated by loss in entropy^17^. For instance, if we add functionality to the ligand that will enable it to engage the protein with an additional hydrogen, the same polar group of the ligand will be hydrogen bonded to water in the unbound state, which is often stronger. Hence the enthalpic gain is minimized.

On the other hand, a major source of entropic gain is derived from desolvation of the ligand when the ligand binds to the protein and hydrophobicity of the ligand helps^17,167^. Kauzmann and others have postulated that entropically unfavorable, ordered water molecules are found near hydrophobic groups of the ligand, in what is referred to as the “iceberg model” where water molecules could form clathrate hydrate structures around hydrophobic groups^168,169^. There are other theories including the “scaled-particle theory” that predicts entropically unfavorable water molecules surrounding the hydrophobic groups^167,170^. These constrained water molecules will be released upon binding to the protein and will contribute significantly to the binding free energy. It has been estimated that transferring a hydrophobic carbon atom from the water to the protein surface contributes ~105 J/mol-Å^2^ to the binding free energy^17,171^. However, it is not possible to increase the hydrophobicity of the ligand after ‘certain’ limit, since it will affect the solubility. Thus, finding the sweet spot between entropy and enthalpy contributors using hydrophobicity is a critical aspect of drug design and remains an open question in the field. We need better NMR methods to quantify desolvation of the ligand.

## Conclusion and future perspectives

As discussed here, NMR has unique strength to provide both static and dynamic views on protein-ligand interactions. NMR experiments are applicable in the drug development stages from initial target validation stage to near clinical lead optimization stages. In addition to the conventional protein small molecule interaction, NMR can handle new modalities such as middle-sized molecules and nucleic acids, which harbors more configurational compared to small molecular ligands. Unlike other available structural strategies, NMR is applicable to a range of conditions. One can expect to apply NMR in cellular conditions to directly monitor the binding of ligands to proteins in cytosol^172^. NMR can also be used to characterize the behavior and interaction in solid-liquid interface including membrane interface^173,174^ as well as in membrane less organelle and phase separation that are relevant for certain disease states^175,176^. This unique ability to directly correlate information from structure and dynamics to thermodynamic and kinetic parameters that dictate protein-ligand interaction makes NMR an invaluable asset in the drug discovery tool kit. Synergistically combining NMR with computational docking methods^177^, molecular dynamics simulations and other biophysical and structural methods including X-Ray crystallography, cryo-electron microscopy^178^, neutron diffraction harbor the potential to provide a clear picture of protein-ligand interactions.

NMR is an indispensable biophysical technique to study conformational ensembles of intrinsically disordered proteins and disordered regions in structured proteins, collectively known as intrinsically disordered regions, IDRs. The disordered regions are prevalent in signaling proteins and transcription factors of eukaryotic cells. The IDRs play a critical role in regulating key cellular processes and are directly implicated in many human diseases, including cancer, cardiovascular disease, neurodegenerative diseases, and diabetes. Given their central role, mutations in the IDR account for one-fifth of all disease-causing mutations^179^. NMR can be useful in designing drugs targeting disordered proteins^180,181^. There a number of examples of IDRs interacting with structured proteins and the IDRs adopt a structure upon complex formation. In some of these cases the IDRs remain dynamic and adopt multiple conformations in the bound state, referred to as a “fuzzy complex”^182^. The fuzzy complexes are examples from nature on how to design a ligand with flexibility and dynamics, in the bound state.

Identifying a lead candidate and optimizing a drug is an arduous endeavor in which a medicinal chemist has to traverse a labyrinth of thermodynamics and kinetic paths to sculpt the ideal molecule. Though we have experimental methods that guide us through this labyrinth, there are still intangibles that we do not fully comprehend. As discussed in this manuscript, solution NMR methods facilitate a wealth of information on the various facets of protein-ligand interactions that help us tease apart the various thermodynamic and kinetic contributions to the binding free energy. Nevertheless, there are blind spots that demand new methods. Even with exquisite details of the energetic landscape, optimizing a molecule is not straightforward and requires an artistic touch. A chemist with a firm grasp of the thermodynamic parameters that dictate the binding event holds a significant advantage and that can make all the difference.

## Figures and Tables

**Figure 5:**
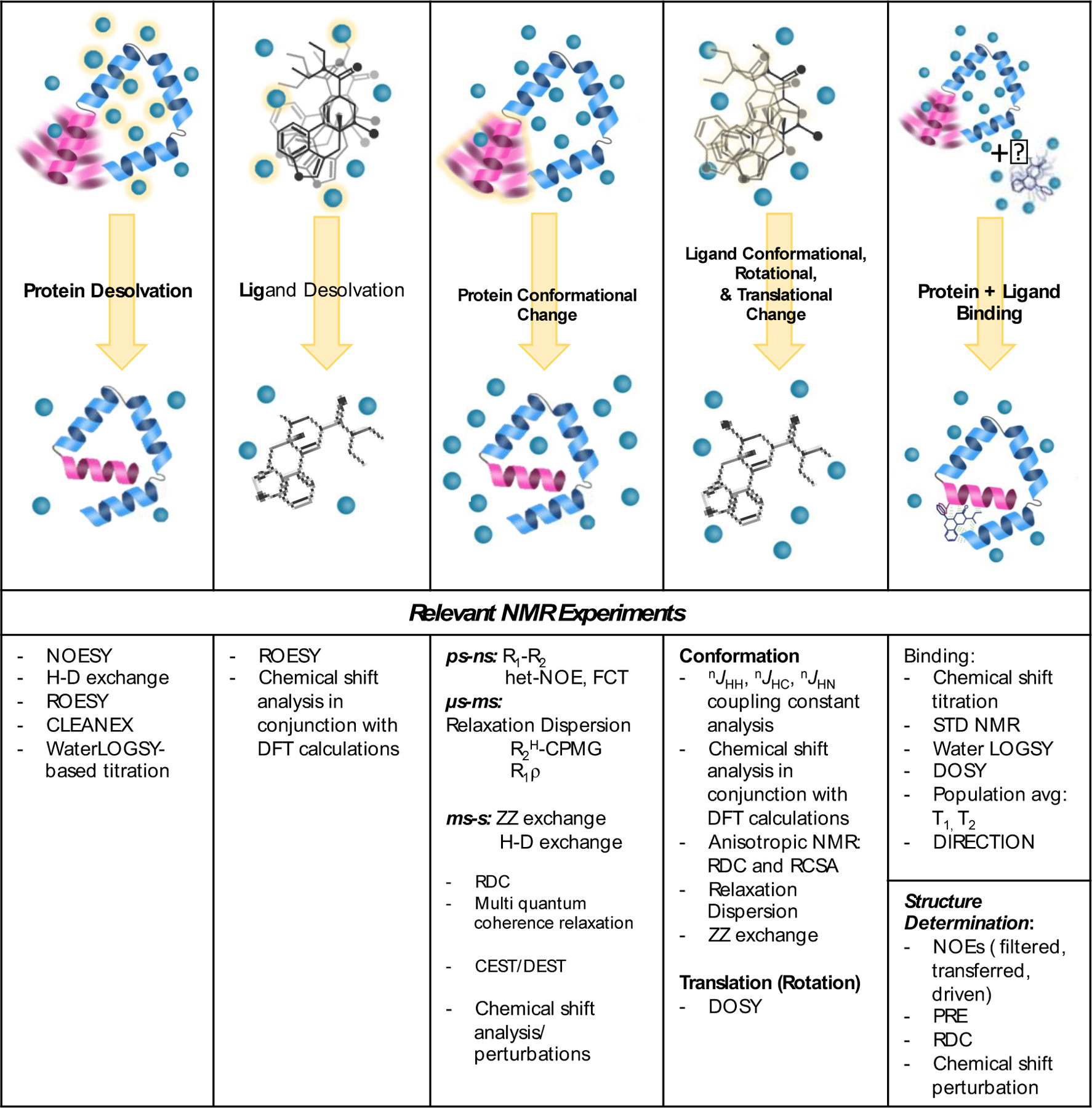
An overview of the different components that contributes binding free energy in typical protein-ligand interaction and the relevant NMR experiments to study these individual contributions. It should be noted that some of methods would not provide absolute measurements of these thermodynamic quantities, rather relative measurements across a panel of lead compounds and their analogs.
